# A potential role for BDNF in recognition behavior revealed through knockout in zebrafish

**DOI:** 10.3389/fnbeh.2026.1710671

**Published:** 2026-04-14

**Authors:** Eleonora Rovegno, Tyrone Lucon-Xiccato, Salvatore D’Aniello, Cristiano Bertolucci, Elia Gatto

**Affiliations:** 1 Department of Life Sciences and Biotechnology, University of Ferrara, Ferrara, Italy; 2 Biology and Evolution of Marine Organisms, Stazione Zoologica Anton Dohrn, Naples, Italy; 3 Department of Chemical, Pharmaceutical and Agricultural Sciences, University of Ferrara, Ferrara, Italy

**Keywords:** behavioral variation, *bdnf*, fish cognition, memory, neurotrophins, zebrafish

## Abstract

**Introduction:**

Brain-derived neurotrophic factor (BDNF) is a key player in the molecular mechanisms underlying learning and memory in mammals. Recent studies have shown that mutant fish lacking BDNF exhibit widespread deficits in learning tasks. Moreover, natural variation in brain BDNF gene expression predicts individual differences in learning ability in fish. These findings suggest that the role of BDNF in cognition may be widespread among vertebrates.

**Methods:**

Following this hypothesis, we used a *bdnf* knockout zebrafish model to investigate whether BDNF is involved in recognition memory in fish. Zebrafish larvae were tested in a novel object recognition test, where their response to a previously encountered stimulus and a novel one was recorded.

**Results:**

Overall, zebrafish did not demonstrate a group-level preference for either stimulus. However, individuals appeared to show variation in their responses to the novel stimulus (either approaching or avoiding it). When accounting for the individual variation, the strength of recognition memory performance was lower in zebrafish lacking *bdnf* compared with control zebrafish, although this effect was influenced by the type of stimulus used. Moreover, the absence of BDNF resulted in less variability in the behavioral response towards the novel stimulus, supporting the role of this protein in shaping individual differences in behavior.

**Discussion:**

Our findings suggest that BDNF may be involved in recognition test performance and the underlying behavior, although the nature of this involvement and the contribution of memory processes remain unclear.

## Introduction

Brain derived neurotrophic factor (BDNF) is a small protein belonging to the neurotrophic growth factor family that has been extensively investigated over the last few decades for its fundamental role in a wide range of neurophysiological processes in the mammalian brain (Leibrock et al., 1989; Numakawa and Kajihara, 2025; Pisani et al., 2023; Toader et al., 2025; Wang et al., 2022). Beyond favoring neuronal proliferation and differentiation (Binder and Scharfman, 2004; Yoshii and Constantine-Paton, 2010), BDNF also enhances synaptic plasticity, promoting learning and memory formation (Bekinschtein et al., 2008; Binder and Scharfman, 2004; Edelmann et al., 2014; Lu et al., 2008). For example, transgenic mice lacking BDNF fail to exhibit long-term potentiation, which is the strengthening of synapses based on recent stimulation (Korte et al., 1998). Consequently, they display poor performance in the Morris water maze task, in which they must remember the spatial position of a safe location (Linnarsson et al., 1997).

The gene coding for BDNF shows sequence conservation across vertebrates and notable similarities appear at the level of structure (Götz et al., 1992). This suggests that its role in cognitive functions may also be conserved. In agreement with this idea, two recent studies demonstrated that BDNF is broadly involved in the cognitive abilities of teleost fish. Adult mutant zebrafish lacking BDNF were unable to acquire a visual associative learning task based on color discrimination and likewise failed to learn a spatial associative task in a T-maze (Lucon-Xiccato et al., 2022). Moreover, at the larval stage, these mutant zebrafish showed reduced habituation learning performance (Lucon-Xiccato et al., 2022). In a second study, natural variation among individuals in the expression of the gene coding for BDNF (*bdnf*) predicted individual differences in visual discrimination learning task in adult zebrafish, with individuals expressing more *bdnf* showing greater performance (Gatto et al., 2025). These works suggest that the role of BDNF in learning processes may be relatively conserved across the vertebrate clade. However, while research in mammals has revealed a broader range of cognitive effects (e.g., Hogan et al., 2021; Hopkins and Bucci, 2010; Papaleo et al., 2011; Seoane et al., 2011; Zhai et al., 2020), in teleost fish the effects of BDNF beyond learning tasks remain largely unexplored.

In this study, we tested whether BDNF is involved in memory in zebrafish. We used a CRISPR/Cas9-generated zebrafish model lacking *bdnf* (*bdnf* ^–/–^; D’Agostino et al., 2022) and a recognition memory task. Recognition memory refers to the ability to spontaneously identify previously encountered stimuli and discriminate them from novel ones without associative learning (Brown et al., 2010; May et al., 2016; Mandler, 1980). The importance of recognition memory extends across various contexts, and it is crucial for adaptive behavior, underpinning essential processes such as foraging, social interactions, predator avoidance, and spatial navigation (Boyle and Brown, 2025). Impairments in recognition memory are also indicative of various neurological and psychiatric conditions (e.g., Imbimbo et al., 2005; Manns and Squire, 1999; Martínez-Drudis et al., 2021; Lobato-Camacho and Faísca, 2024; Trivedi, 2006). Critically, several studies in mammals have revealed that BDNF is involved in recognition memory (Brivio et al., 2020; Hopkins and Bucci, 2010; Seoane et al., 2011; Zhai et al., 2020).

The recognition memory task we adopted in our study is often referred to as the Novel Object Recognition test (NORt). It was originally developed for rodents (Ennaceur and Delacour, 1988), but is now widely used in a range of species (Hamilton, 2018), including other mammals (Golden and Dilger, 2025; Piotti et al., 2022; Visser et al., 2002; Zola et al., 2000), birds (Damphousse et al., 2022; Mettke-Hofmann et al., 2002), amphibians (Kundey and Phillips, 2021), and fish (Braida et al., 2014; Lucon-Xiccato and Dadda, 2014, 2016; May et al., 2016; Wallace and Hofmann, 2021). The procedure involves a familiarization phase where the subject explores two identical objects, followed by a test phase in which one of the objects is replaced with a novel one. The differential response elicited by the familiar and the novel object in the test phase serves as a measure of recognition memory.

To investigate the role of BDNF in recognition memory, we applied the NORt in zebrafish *bdnf ^–/–^*, using their wild-type siblings (*bdnf ^+/+^*) as controls. We performed the NORt in larval fish, because, as in birds and mammals (Pascalis and de Schonen, 1994; Pandey et al., 2024; Reger et al., 2009), zebrafish already exhibit traces of object recognition memory during early development (Bruzzone et al., 2020; Gatto et al., 2022a; Gatto et al., 2022b). Moreover, the use of larvae allowed us to test a relatively large number of subjects, thereby increasing our statistical power. We predicted that if BDNF is involved in the neural processes underlying recognition memory, *bdnf ^–/–^* larvae would display impairments in the NORt.

## Materials and methods

### Experimental subjects

Adult mutant zebrafish of the *bdnf ^–/–^* line (D’Agostino et al., 2022) as well as their wild-type siblings (*bdnf ^+/+^*) were housed and maintained at the facility of the University of Ferrara. The null mutant line was generated through a CRISPR/Cas9-mediated deletion of 40 bp in *bdnf* coding sequence that affects all open reading frame gene isoforms. The mutation did not affect survival, reproductive success or morphology of the *bdnf ^–/–^* line (D’Agostino et al., 2022). The two zebrafish strains were housed separately in 50 L glass tanks (60 × 40 × 40 cm) under a 14:10 light:dark photoperiod and temperature of 27 ± 1°C. A mechanical, biological and chemical filtration system kept optimal water conductivity (606.7 ± 60.18 μS/cm), nitrite below 0.1 mg/L and nitrate below 50 mg/L. Zebrafish were fed two/three times per day with live brine shrimp (*Artemia salina*) nauplii and dry food (Staple food Vipan, Sera, Heinsberg, Germany).

The subjects for the experiments (*N* = 108; 54 *bdnf ^–/–^* and 54 *bdnf ^+/+^* zebrafish) belonged to a *bdnf ^–/–^* and a *bdnf ^+/+^* line originally derived from siblings obtained with *bdnf ^–/–^* × *bdnf ^+/+^* crosses. We used larvae as subjects, generated from separate *bdnf ^–/–^* × *bdnf ^–/–^* and *bdnf ^+/+^* × *bdnf ^+/+^* crosses. The choice of using larvae as subjects was due to logistical reasons: given the known variability in fish cognition and behavior, we aimed to test a relatively large sample size, which can be obtained more efficiently by studying larvae directly rather than waiting for a sufficient number of individuals to develop into adults. Eggs were collected in several petri dishes (Ø 10 cm, h:1.5 cm) in a solution of E3 1x (5 mM NaCl, 0.17 mM KCl, 0.33 mM CaCl_2_, 0.33 mM MgSO_4_) and Methylene blue (0.0016 g/l). Hatched larvae (3 day-post fertilization, dpf) were transferred in fish water (50 × : 25 g Instant Ocean, 39.25 g CaSO4 and 5 g NaHCO3 for 1 l) and kept in a 12:12 LD cycle at 28°C. Larvae were fed from 5 dpf with powdered food with phytoplankton (51% spirulina) and zooplankton (18% krill; Sera Micron Nature).

### Novel object recognition test

**FIGURE 1:**
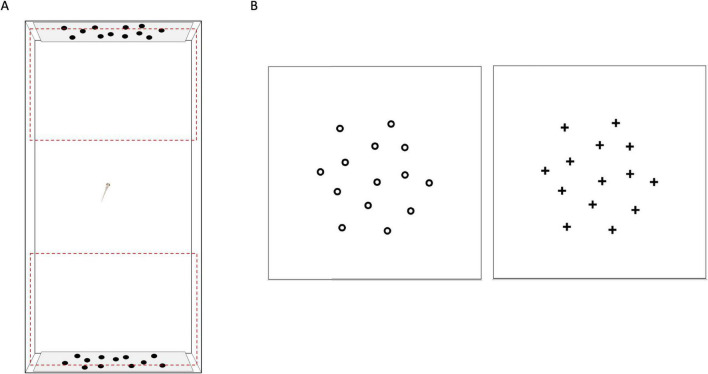
Representation of the experimental apparatus and stimuli used. **(A)** Top view of experimental apparatus during familiarization phase. Lateral sectors in which time close to the stimuli was scored are outlined with dashed red lines. **(B)** Stimuli used: array of circles stimulus on the left, array of crosses stimulus on the right.

The NORt procedure followed the one previously used in zebrafish larvae (Bruzzone et al., 2020; Gatto et al., 2022a). At 14 dpf, each individual larva was transferred with a Pasteur pipette into an experimental apparatus (8 × 4 × 5 cm) filled with 90 mL of fish water (Figure 1A). The apparatus was illuminated from above by LED strips (natural daylight, 6,000 K, ELCART, Italy). In this apparatus, the visual stimuli were presented along the short sides (Figure 1A). The stimuli used were white panels with printed arrays of 12 identical black figures, either crosses or circles, each 1.5 × 1.5 mm, as described in Gatto et al., 2022a (Figure 1B).

After being introduced into the apparatus, the larvae were exposed for 1 h to two identical copies of the same stimulus for familiarization (Gatto et al., 2022a). The stimulus for the familiarization (i.e., crosses or circles) was randomized among subjects, with care to ensure an equal number of subjects per strain with the same familiarization stimulus. Immediately after the familiarization phase, each larva was moved into a new experimental apparatus, identical to the first one except for the stimuli presented: one being the familiar stimulus and the other being the novel stimulus. In practice, subjects that familiarized either with the crosses or with the circles were here exposed to one stimulus with the crosses and one stimulus with the circles. The test phase lasted 15 min. Subject manipulation between the two phases lasted a few seconds, as the experimental apparatuses for the test were placed next to the familiarization ones before the transfer (i.e., May et al., 2016; Lucon-Xiccato et al., 2020). All the apparatuses were cleaned and the water was changed after the subjects have completed the test. Both the familiarization phase and the test phase were video recorded from above with cameras (HDR-CX405, Sony Europe B.V., Weybridge, United Kingdom), placed 1 m above the experimental tanks.

### Data scoring

Three video recordings were discarded due to technical issues (total analyses video: 51 *bdnf ^–/–^*, 54 *bdnf ^+/+^*). An experimenter blind to the aim of the study and the subjects’ genotype analyzed the video-recordings with the manually operated chronometer software Ciclic timer (version 1.3). By superimposing a template to the video-recordings played back on a computer monitor, the experimenter virtually divided each apparatus into three identical sectors: one central and two lateral sectors facing the stimuli (Figure 1A). Then, the experimenter scored the time spent in each sector during the familiarization and test phases. For the familiarization phase, we considered the first and last 15 min to assess the exploratory pattern of larvae in the novel environment and evaluate potential side biases, obtaining observations comparable in length to those of the test phase. In addition to the raw time spent in the different sectors, we calculated a series of proportion indices useful to better describe the behavior of the subjects.

From the familiarization phase data, we computed the proportion of time spent in one of the two lateral sectors as (time in one lateral sector/total time spent in the two lateral sectors). From the recordings of the test phase, we assessed memory performance of the subjects by calculating two indices. The first was a relative recognition memory index calculated as [(time in the novel stimulus sector - time in the familiar stimulus sector)/(time in the novel stimulus sector + time in the familiar stimulus sector)] (Akkerman et al., 2012; Ennaceur et al., 2005). The second index was used to control for the fact that the response to novelty exhibited by individual subjects is highly variable and dependent upon many factors (e.g., object features, individual experience, sex, age). Indeed, previous studies in mammals (Antunes and Biala, 2012) and fish, both adults (Braida et al., 2014; Gerlai, 2016; Lucon-Xiccato and Dadda, 2014; Magyary, 2019; May et al., 2016) and immature (Bruzzone et al., 2020; Gatto et al., 2022b), showed that some individuals tend to approach novel stimuli (neophilic response), while others avoid them (neophobic response). The second index aligned the preference/avoidance of the larvae on the same direction. This absolute recognition memory index was calculated as | relative recognition memory index | . The two indices represent the behavioral response elicited by the novel stimulus and the strength of the animal’s discrimination ability, respectively.

### Statistical analysis

Statistical analyses were performed in RStudio version 4.2.2.^1^ Descriptive statistics were reported as mean ± standard deviation and the significance threshold for the statistical tests was set at *p* < 0.05.

For the familiarization phase, we firstly compared the attraction toward the stimuli displayed by the two strains considering the total amount of time spent in the lateral sectors with two samples *t*-test. We performed the same analysis on the first and the last 15 min of the familiarization phase. Then, we controlled for potential side biases at population level due to the experimental tank by assessing whether the proportion of time spent in one, arbitrarily chosen, of the two lateral sectors significantly differed from 0.5 with a one-sample *t*-test. To test for difference in an eventual side bias between strains, we also analyzed the proportion of time spent in one of the two lateral sectors using a linear mixed-effect model (LMM, “*lmer”* function) fitted with strain as fixed effect, and the trial as a random effect to account for the hierarchical structure of the data in our experimental design. We also include in the model the stimulus type (crosses vs. circles) as fixed factor and its interaction with strain. To deal with the variability displayed by subjects in the amount of time spent exploring the two stimuli, the dependent variable was weighted to the overall time spent in the lateral sectors. Because the random effects did not explain any variance in the initial linear mixed-effect model (singularity fit issue), we removed it from the final model. We then performed a generalized linear model with Gaussian distribution without including any random effects. We used this approach for analyzing the data of the first and last 15 min of familiarization phase. Significance of the model’s parameters was assessed via the “*Anova*” function from the “car” R package.

In the test phase, we used a similar approach. We first compared the total time spent by subjects of the two strains near both stimuli using a two-sample *t*-test. Due to the side bias observed in the first 15 min of the familiarization phase, we investigated whether this side bias could affect the assessment of subjects’ recognition memory ability. To this goal, we analyzed the proportion of time spent in the same lateral sectors of the experimental tanks using a generalized mixed-effect model with Gamma distribution (link = “inverse”) fitted with strain and the experimental phase (first part of the familiarization phase vs. test phase) and the interaction of these two variables as fixed factors, and subject ID as random effect. The response was weighted for the total amount of time spent in both lateral sectors. The initial model was fitted also with trial as a random effect, but this term did not explain any variance in the model (singularity fit issue), so it was removed from the final model. We also conducted a Spearman rank correlation analysis between the time spent in the sector in the familiarization phase (initial 15 min) and in the test phase.

Then, we focused the analysis on the recognition memory index. We used a one sample *t*-test to test whether the subjects from each strain displayed a general tendency to avoid or approach the novel stimulus over the familiar one by testing the relative recognition memory index against the null choice (null choice index = 0).

Individual differences in the relative recognition memory index were graphically compared between the two strains. We assessed differences in the variance of the memory index between the two strains using the reweighted Wasserstein distance of the Anderson-Darling test (“*dts_test*” function from the “*twosamples*” R package) and we analyzed the distribution shape with kurtosis and skewness calculation (“*moments*” R package, and Shapiro-Wilk test for normality). We also compared the occurrence of individuals with markedly low relative recognition memory indices between strains using a Chi-square test. These individuals were identified using a threshold corresponding to the first significant gap in the distribution (i.e., one of the 10% largest gaps), starting from the center of the distribution.

To assess differences in the relative recognition memory index between strains we used a LMM fitted with strain and stimulus type as fixed effect and trial as random effect. Also in this case, the response variable was weighted for the total amount of time spent in exploring the novel and familiar stimulus. Due to a singularity fit issue in the model that included the random effects (trial), we re-ran the analysis by removing this term and performing a generalized linear model with Gaussian distribution. The relative recognition memory index expressed by the two strains was further studied using graphical representations of the sample distribution.

We then used a one tailed *t*-test to test whether the absolute recognition memory index significantly greater than zero, an analysis that would help to detect the ability to discriminate the stimuli even if subjects showed different neophilic or neophobic reactions. Strain differences in the absolute recognition memory index were investigated with a generalized mixed-effect model with Gamma distribution (link = “inverse”) fitted with strain and stimulus type as fixed effects and trial as a random effect. The response was weighted for the total amount of time spent in exploring the novel and familiar stimulus. Significance of the model’s parameters was assessed via the “*Anova*” function from the “car” R package.

Zebrafish larvae often display reduced mobility or diminished responsiveness to stimuli, behaviors that could bias the results of a preference-based test such as the one used in our study. Therefore, we conducted an initial behavioral screening to ensure that our statistical inference was based only on individuals that were actively engaging with the task. As a threshold, we required that subjects explore both lateral sectors during the familiarization phase, and we included only those that met this criterion. This procedure ensured that the analyses were performed on fish that had actually detected both stimuli. A total of 24 out of 105 larvae (22.86%) showed reduced activity and did not meet the criterion of visiting both sectors (14 *bdnf ^–/–^* and 10 *bdnf ^+/+^*). These individuals were removed from the dataset, resulting in a final sample size of 81 subjects (37 *bdnf ^–/–^* and 44 *bdnf ^+/+^*). There was no significant difference between strains in the occurrence of these subjects with reduced activity (Chi-squared test: χ^2^_1_ = 1.187; *p* = 0.276).

## Results

### Familiarization phase

The subjects from the two strains spent a similar amount of time exploring the two copies of the stimulus in the first 15 min of the familiarization phase (two sample *t*-test: *t*_79_ = 1.009, *p* = 0.316), with the *bdnf ^–/–^* subjects exploring the stimuli during 68.50 ± 8.60% of the time and *bdnf ^+/+^* subjects during 66.54 ± 8.80% of the time. Similarly, the two strains did not differ in the amount of time spent exploring the stimuli in the last 15 min of the familiarization phase (*bdnf ^–/–^* = 71.87 ± 7.69%; *bdnf ^+/+^* = 72.53 ± 9.97%; *t*_79_ = 0.328, *p* = 0.744), suggesting that both strains equally familiarized with the stimuli. Overall, in the first 15 min the subjects showed a bias toward one of the two sides of the familiarization apparatus (one sample *t*-test: t_80_ = 3.109, *p* = 0.003). However, this side bias was not affected by the strain and by the stimulus type (two-ways ANOVA: strain: χ^2^_1_ = 1.547, *p* = 0.214; stimulus type: χ^2^_1_ = 0.443, p = 0.506; strain × stimulus type: χ^2^_1_ = 0.270, *p* = 0.604; Supplementary Table 1). Moreover, the side bias disappeared during the familiarization: when looking at the last 15 min of this phase, the side preference did not significantly differ from 0.5 (*t*_80_ = 1.534, *p* = 0.129) and was not affected by the stimulus type or the strain of the larvae (strain: χ^2^_1_ = 0.001, *p* = 0.981; stimulus type: χ^2^_1_ = 0.282, p = 0.595; strain × stimulus type: χ^2^_1_ = 0.320, *p* = 0.572; Supplementary Table 2).

### Test phase

#### Side bias

During the test phase, the two strains spent a similar amount of time exploring the two stimuli (sum of time spent near the novel and the familiar stimulus; *bdnf ^–/–^*: 72.43 ± 7.99%; *bdnf ^+/+^*: 72.90 ± 7.42%; Two sample *t*-test: *t*_79_ = 0.277, *p* = 0.782). The time spent in one lateral sector in the initial 15 min of the familiarization phase was significantly different from that of the test phase (χ^2^_1_ = 4,984.061, *p* < 0.001; Supplementary Table 3). Moreover, there was no significant correlation between these two variables (Spearman rank Correlation: ρ = 0.013, S = 87,450, *p* = 0.912). This suggested that the behavior of the subjects in the test phase was not affected by the side bias observed in the familiarization phase.

#### Relative recognition memory index

**FIGURE 2:**
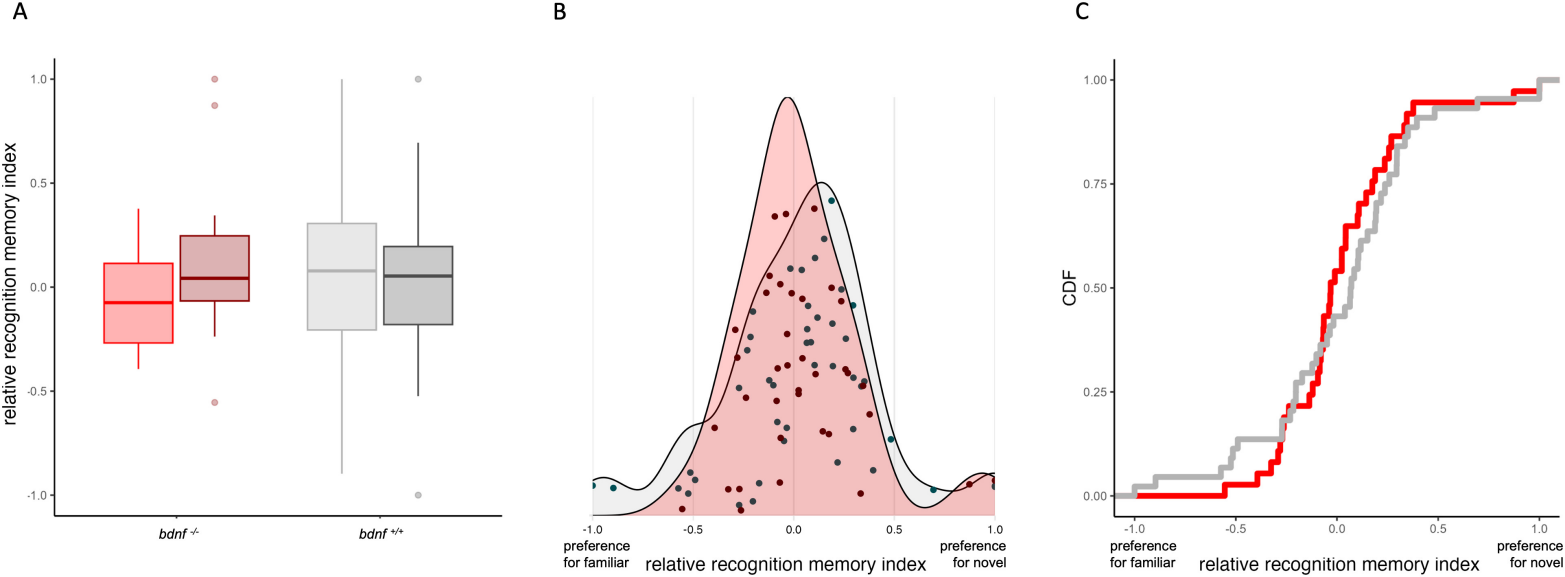
Graphical representations of the relative recognition memory index expressed by the *bdnf^+/+^* (gray) and *bdnf ^– /–^*(red) larvae. **(A)** Boxplot showing the relative recognition memory index of the two strains, divided by type of novel stimulus (light color: arrays of crosses; dark color: arrays of circles). **(B)** Density distribution plots divided by strain; individual datapoints are overlaid to show the values of each subject. **(C)** Cumulative distribution plot illustrating the proportion of subjects below each value of the recognition memory index; steeper portions of the curve indicate ranges where many individuals cluster, while flatter regions correspond to gaps or sparsely populated areas of the distribution.

We first analyzed recognition memory using a relative index that quantified the preference for the novel versus the familiar stimulus across all subjects within each line. The relative recognition memory index did not significantly differ from zero in both the *bdnf ^+/+^* (one sample *t*-test: *t*_43_ = 0.472, *p* = 0.639) and the *bdnf ^+/+^* zebrafish (*t*_36_ = 0.585, *p* = 0.563), indicating the absence of an average preference for one of the stimuli. The mixed-effects analysis on the relative recognition memory index found no effect of the strain (χ^2^_1_ = 0.003, *p* = 0.959), the stimulus type (χ^2^_1_ = 0.491, *p* = 0.483) or the interaction (χ^2^_1_ = 1.631, *p* = 0.202; Figure 2A and Supplementary Table 4).

The comparison of variance in the recognition memory index found not average difference between strains (Anderson-Darling test via DTS function: DTS = 2.262, *p* = 0.488). However, there were clear differences in the shape of the distributions between the two strains. The index distribution in the *bdnf ^–/–^* group was highly skewed (positive skew: 1.148) and significantly deviated from normality (Shapiro–Wilk test: *W* = 0.914, *p* = 0.008). In contrast, the distribution in the *bdnf ^+/+^* group did not differ significantly from a normal distribution (*W* = 0.963, *p* = 0.173) and showed leptokurtosis (kurtosis: 3.905), indicating a sharp central peak with extreme values symmetrically represented in both tails (Figure 2B). Among the seven individuals showing a stronger neophobic response to the novel stimulus (left side of the distribution; Figure 2A), six were *bdnf ^+/+^* and one was *bdnf ^–/–^* (Chi-square test: χ^2^_1_ = 3.571, *p* = 0.059). This observation was confirmed by a cumulative distribution plot divided by strain (Figure 2C): the *bdnf ^+/+^* subjects showed a higher probability of observing datapoints at lower values of the index (i.e., values toward -1), corresponding to a preference for the familiar stimulus.

#### Absolute recognition memory index

**FIGURE 3:**
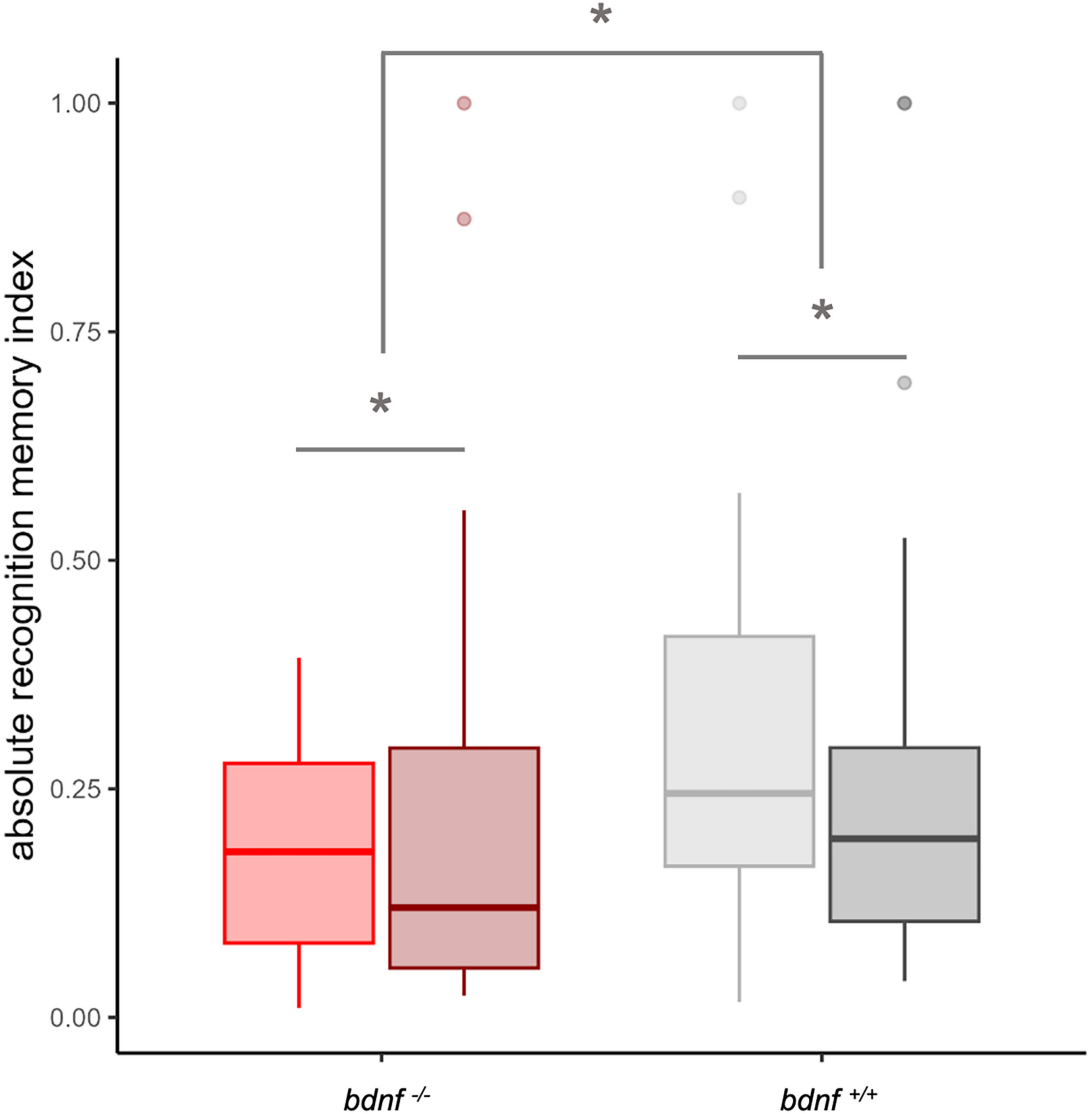
Boxplot representing the absolute recognition memory index of the two strains (*bdnf ^+/+^*: grey; *bdnf ^– /–^*: red), divided by type of novel stimulus (light color: arrays of crosses; dark color: arrays of circles). Asterisks indicated significant differences between strain (*bdnf ^+/+^* vs. *bdnf ^– /–^* red) and within strain accordingly to the type of stimulus used for the familiarization phase.

To account for the fact that individual animals may respond differently to the same stimulus, either approaching or avoiding it, we also analyzed an absolute recognition memory index. This aligned the preferences of all subjects in the same direction and, while lacking the ability to detect population-level preferences, allowed us to compare the intensity of memory across subjects showing high variability in their responses to the stimuli. The absolute recognition memory index significantly differed from zero in both strains (one tailed *t*-test; *bdnf ^+/+^*: *t*_43_ = 77.451, *p* < 0.001; *bdnf ^–/–^*: *t*_36_ = 6.006, *p* < 0.001). The absolute recognition memory index was significantly affected by the strain (χ^2^_1_ = 754.118, *p* < 0.001; Figure 3 and Supplementary Table 5): the *bdnf ^+/+^* subjects showed greater index (0.30 ± 0.27) than the *bdnf ^–/–^* subjects (0.22 ± 0.22). The analysis did not reveal a significant effect of stimulus on the absolute recognition memory index (χ^2^_1_ = 2.105, *p* = 0.147), but a significant interaction strain × stimulus type (χ^2^_1_ = 456.366, *p* < 0.001). This suggested a possible influence of the type of stimulus on the strength of preference between subjects from the two strains (Figure 3).

## Discussion

BDNF is considered a key element of the molecular mechanisms underlying memory formation in mammals (Jain, 2021; Wang et al., 2022). Based on sequence and structural similarities (Götz et al., 1992) as well as phenotypic evidence (Lucon-Xiccato et al., 2022), we hypothesized that BDNF may play a similar role in teleost fish. To test this, we investigated recognition memory in *bdnf*-knockout zebrafish larvae using the novel object recognition test. Our findings provide evidence that BDNF contributes to performance in the object recognition test; however, it remains unclear whether this contribution was mediated by memory processes or by behavioral mechanisms.

Bruzzone et al. (2020) and Gatto et al. (2022a) reported that zebrafish larvae at 14 dpf may be able to discriminate between novel and familiar stimuli. We did not find clear evidence of this capacity: overall, our sample did not show an average preference for either the novel or the familiar stimulus, as indicated by a relative memory index that did not statistically differ from zero. Moreover, no effect of *bdnf* loss was detected when considering this relative memory index. However, we observed substantial individual variation in how larvae approached the task, with some subjects showing neophobia and others showing neophilia toward the novel stimulus. This variation may explain the lack of a significant relative index in the present study. Importantly, after correcting the memory index for individual differences in the approach to the novel stimulus, we found statistical evidence suggesting that *bdnf ^–/–^* larvae may display weaker recognition memory compared to wild-type larvae. This effect was however modulated by the type of stimulus. Although in the familiarization phase the larvae appeared to respond similarly to the two types of stimuli, in the test phase, when both stimuli were presented simultaneously, one stimulus may have intrinsically attracted or repelled the zebrafish more than the other based on its perceptual characteristics.

We believe that our results should be interpreted with caution. On the one hand, one might conclude that BDNF plays a role in memory in zebrafish, albeit marginal as suggested by the absence of detectable effects on one of the two indices of recognition memory. On the other hand, it is possible that the effect detected with the absolute index was largely driven by behavioral differences in how fish from the two lines approached the stimuli, or by preferences for specific objects, rather than by actual differences in memory. Overall, the presence and extent of BDNF involvement in zebrafish recognition memory remain difficult to clarify based on our study.

While this remains speculative, one may be tempted to conclude that, if BDNF plays a role in zebrafish recognition memory, its contribution is likely minimal; otherwise, clearer differences between the two lines compared in this study would have been observed. Conversely, BDNF contributes importantly to recognition memory at multiple levels in mammals (Brivio et al., 2020; Hopkins and Bucci, 2010; Seoane et al., 2011; Zhai et al., 2020). For instance, in mice and rats, information retrieval during the novel object tests increases hippocampal BDNF levels, and blocking BDNF signaling impairs object memory (Radiske et al., 2017). Both shared features and notable differences may exist in the role of BDNF between mammals and fish across memory functions. Investigating what determines this potential difference between mammals and fish would be valuable. One aspect worth attention is that, in fish, neurotrophins have diversified through genome duplication, resulting in a set of neurotrophins that differs from that of tetrapods (Hallböök, 1999) and may be involved in memory processes. Moreover, considering that the most pronounced cognitive impairments in the absence of BDNF were observed in adult zebrafish (Lucon-Xiccato et al., 2022), the limited effects observed in the present study could be related to the developmental stage of the subjects. It therefore seems valuable to provide additional data on adult zebrafish and recognition memory to further investigate the role of BDNF.

However, before accepting the two biological interpretations above, methodological considerations should be taken into account. The difficulty in detecting an effect in our study might partly arise from the methodological complexity of measuring recognition memory through novelty-response tests in larvae. This complexity is highlighted by the lack of a significant relative memory index, and probably attributable to the coexistence of both neophobic and neophilic individuals in our otherwise relatively homogeneous sample. We therefore conclude that the role of BDNF in memory in zebrafish larvae should also be investigated using complementary approaches that are less sensitive to the influence of individual behavioral differences. As mentioned before, using adult zebrafish, and thus trading some statistical power for a model that allows characterization of more complex behaviors, may also be relevant in this context.

Variation in behavior toward the stimuli also highlighted an interesting result. Individual differences in novelty preference in fish are often not considered random measurement errors but a continuum of stable behavioral differences within the population (Magurran, 1986; Toms et al., 2010) with biological significance (e.g., Brown et al., 2013). Due to their stability, these differences are often referred to as personality traits and tend to covary, forming so-called behavioral syndromes (Castanheira et al., 2013; Franks et al., 2023; Toms and Echevarria, 2014). For example, neophilic fish are often more explorative, bolder, and more active than neophobic fish (Martins et al., 2012; Lucon-Xiccato et al., 2019). Moreover, more recent research has documented individual differences in cognition in fish (reviewed in Lucon-Xiccato and Bisazza, 2017). Based on this literature, we suggest that the variation in the relative index observed in our study, while probably representing a confounding factor in our main analyses, may nevertheless have biological significance, reflecting individual differences in novelty preference and/or memory. If this interpretation was correct, it is interesting that larvae lacking BDNF showed marginally less behavioral variability, with a lower occurrence of neophobic individuals. Although this requires direct experimental investigation, we suggest that BDNF may be involved in individual differences in memory or novelty preference, alongside other genes that have been recently studied (Baker and Wong, 2021; Chen et al., 2015; Rovegno et al., 2024, 2025). In support of this idea, in recent studies on adults we found a role of BDNF in shaping individual differences in learning (Gatto et al., 2025) and behaviors normally associated with personalities (Lucon-Xiccato et al., 2023). Moreover, in humans, variation in BDNF due to polymorphisms and other factors have been shown to affect personality traits, such as anxiety (Freundlieb et al., 2015; Kazantseva et al., 2015; Lang et al., 2005; Minelli et al., 2011; Montag, 2014). Last, while BDNF was not considered in these studies, there is evidence that animal personalities may depend on neural plasticity (Øverli and Sørensen, 2016). Therefore, while the direct link and underlying mechanisms are not yet fully understood, a role for BDNF in shaping individual differences in zebrafish larval behavior seems entirely plausible.

In conclusion, our research provides evidence for a role of BDNF in object recognition in zebrafish larvae. However, given the confounds related to individual differences in behavior and potential biases toward specific stimuli, it remains unclear whether this effect was driven by memory or by other behavioral mechanisms.

## Data Availability

The raw data supporting the conclusions of this article will be made available by the authors, without undue reservation.

## Footnotes

1
https://www.R-project.org/
