# Supplementary material for: A potential role for BDNF in recognition behavior revealed through knockout in zebrafish
Source: Front Behav Neurosci. 2026 Apr 14;20:1710671. doi: 10.3389/fnbeh.2026.1710671 (PMC13121150; doi:10.3389/fnbeh.2026.1710671)
Supplement: Supplementary file 1 [file Supplementary_file_1.pdf]

Supplementary Materials

Table 1. Output from linear model testing the effect of type of stimulus, strain and their interaction on the percentage of time individuals spent in one of the two lateral sectors during the first 15 minutes of the familiarization phase.

Fixed Effects					
	Estimates	SE	95% CI	t	p
Intercept	0.52	0.03	0.47 – 0.58	19.10	<0.001
type of stimulus [arrays of circles]	0.00	0.04	-0.07 – 0.08	0.07	0.941
strain [*bdnf*$^{+/+}$]	0.02	0.04	-0.06 – 0.09	0.47	0.639
type of stimulus [arrays of circles] × strain [*bdnf*$^{+/+}$]	0.03	0.05	-0.07 – 0.13	0.52	0.604
Model fit					
N° observation	81				
R^2	0.030				
Model equation: response ~ strain × type of stimulus, weights = total time spent towards both lateral sectors<br>Response: percentage of time spent in one of the two lateral sectors over the total time spent in both sectors during the first 15 minutes of the familiarisation phase					

Table 2. Output from linear model testing the effect of type of stimulus, strain and their interaction on the percentage of time individuals spent in one of the two lateral sectors during the last 15 minutes of the familiarization phase.

Fixed Effects					
	Estimates	SE	95% CI	t	p
Intercept	0.52	0.03	0.46 – 0.59	15.42	<0.001
type of stimulus [arrays of circles]	0.00	0.05	-0.09 – 0.10	0.06	0.953
strain [*bdnf*$^{+/+}$]	0.02	0.05	-0.07 – 0.11	0.43	0.670
type of stimulus [arrays of circles] × strain [*bdnf*$^{+/+}$]	-0.04	0.06	-0.16 – 0.09	-0.57	0.572
Model fit					
N° observation	81				
R^2	0.010				

Model equation: response ~ strain × type of stimulus, weights = total time spent towards both lateral sectors

Response: percentage of time spent in one of the two lateral sectors over the total time spent in both sectors during the last 15 minutes of the familiarisation phase

Table 3. Output from the general linear mixed-effect model with Gamma distribution testing the effect of experimental phase, strain and their interaction on the percentage of time individuals spent in the same lateral sector.

Fixed Effects					
	Estimates	SE	95% CI	t	p
Intercept	6.93	0.60	5.85 – 8.23	22.40	<0.001
experimental phase [first 15-minutes familiarisation]	0.94	0.00	0.93 – 0.94	-19.83	<0.001
strain [*bdnf*$^{+/+}$]	1.04	0.12	0.82 – 1.31	0.30	0.761
experimental phase [first 15-minutes familiarisation] × strain [*bdnf*$^{+/+}$]	0.85	0.00	0.84 – 0.86	-36.50	<0.001
Random Effects					
σ					10.38
τ_{00}					4.01
ICC					0.28
N° $_{subject}$					81
Model fit					
N° $_{observation}$					162
Marginal R^2					0.001
Conditional R^2					0.279

Model equation: response ~ phase × strain + (1|subject), family=Gamma(link=inverse), weights = total time spent towards lateral sectors

Response: percentage of time spent in the same lateral sector over the total time spent in both sectors

σ = within-group variance; τ_{00} = between-group variance; ICC = interclass correlation coefficient

Table 4. Output from linear model testing the effect of type of stimulus, strain and their interaction on the relative recognition memory index.

Fixed Effects					
	Estimates	SE	95% CI	t	p
Intercept	-0.06	0.09	-0.23 – 0.11	-0.72	0.470
type of stimulus [arrays of circles]	0.17	0.12	-0.07 – 0.41	1.41	0.157
strain [*bdnf*$^{+/+}$]	0.12	0.12	-0.12 – 0.35	0.97	0.332
type of stimulus [arrays of circles] × strain [*bdnf*$^{+/+}$]	-0.21	0.16	-0.53 – 0.11	-1.28	0.202
Model fit					
N° observation	81				
R^2	0.027				
Model equation: response ~ phase × strain, weights = total time spent towards both lateral sectors<br>Response: relative recognition memory index					

Table 5. Output from the general linear mixed-effect model with Gamma distribution testing the effect of type of stimulus, strain and their interaction on the absolute recognition memory index.

Fixed Effects					
	Estimates	SE	95% CI	t	p
Intercept	145.77	46.93	76.76 – 276.83	15.47	<0.001
type of stimulus [arrays of circles]	0.37	0.02	0.33 – 0.41	-18.92	<0.001
strain [*bdnf*$^{+/+}$]	0.18	0.01	0.16 – 0.20	-34.16	<0.001
type of stimulus [arrays of circles] × strain [*bdnf*$^{+/+}$]	3.77	0.23	3.33 – 4.26	21.36	<0.001
Random Effects					
σ	530.26				
τ_{00}	270.31				
ICC	0.34				
$N°_{trial}$	5				
Model fit					
$N°_{observation}$	81				
Marginal R^2	0.000				
Conditional R^2	0.338				
Model equation: response ~ phase × strain + (1|trial), family=Gamma(link=inverse), weights = total time spent towards lateral sectors<br>Response: absolute recognition memory index					

σ = within-group variance; τ_{00} = between-group variance; ICC = interclass correlation coefficient
